# Role of MicroRNA-143 in Nerve Injury-Induced Upregulation of Dnmt3a Expression in Primary Sensory Neurons

**DOI:** 10.3389/fnmol.2017.00350

**Published:** 2017-11-09

**Authors:** Bo Xu, Jing Cao, Jun Zhang, Shushan Jia, Shaogen Wu, Kai Mo, Guihua Wei, Lingli Liang, Xuerong Miao, Alex Bekker, Yuan-Xiang Tao

**Affiliations:** ^1^Department of Anesthesiology, Rutgers New Jersey Medical School, The State University of New Jersey, Newark, NJ, United States; ^2^Department of Anesthesiology, General Hospital of Guangzhou Military Command, Guangzhou, China; ^3^Neuroscience Research Institute, College of Basic Medicine, Zhengzhou University, Zhengzhou, China; ^4^Department of Anesthesiology, Union Medical Center, Tianjin, China; ^5^Department of Anesthesiology, Yantai Affiliated Hospital of Binzhou Medical University, Yantai, China; ^6^Departments of Cell Biology & Molecular Medicine and Physiology, Pharmacology & Neuroscience, Rutgers New Jersey Medical School, The State University of New Jersey, Newark, NJ, United States

**Keywords:** miR-143, Dnmt3a, *Oprm1*, dorsal root ganglion, neuropathic pain

## Abstract

Peripheral nerve injury increased the expression of the DNA methyltransferase 3A (*Dnmt3a*) mRNA and its encoding Dnmt3a protein in injured dorsal root ganglia (DRG). This increase is considered as an endogenous instigator in neuropathic pain genesis through epigenetic silencing of pain-associated genes (such as *Oprm1*) in injured DRG. However, how DRG DNMT3a is increased following peripheral nerve injury is still elusive. We reported here that peripheral nerve injury caused by the fifth spinal nerve ligation (SNL) downregulated microRNA (miR)-143 expression in injured DRG. This downregulation was required for SNL-induced DRG Dnmt3a increase as rescuing miR-143 downregulation through microinjection of miR-143 mimics into injured DRG blocked the SNL-induced increase in Dnmt3a and restored the SNL-induced decreases in *Oprm1* mRNA and its encoding mu opioid receptor (MOR) in injured DRG, impaired spinal cord central sensitization and neuropathic pain, and improved morphine analgesic effects following SNL. Mimicking SNL-induced DRG miR-143 downregulation through DRG microinjection of miR143 inhibitors in naive rats increased the expression of Dnmt3a and reduced the expression of *Oprm1* mRNA and MOR in injected DRG and produced neuropathic pain-like symptoms. These findings suggest that miR-143 is a negative regulator in Dnmt3a expression in the DRG under neuropathic pain conditions and may be a potential target for therapeutic management of neuropathic pain.

## Introduction

Nerve injury-induced neuropathic pain causes prolonged suffering and significantly decreases the quality of life in patients. Conventional analgesics, such as non-steroidal anti-inflammatory drugs and opioids, are poorly effective in the treatment of this disorder, as most of them provide non-specific symptomatic relief in regards to the cause of neuropathic pain (Vorobeychik et al., 2011; Dworkin et al., 2013). Neuropathic pain is characterized by spontaneous ongoing pain, or intermittent pain, hyperalgesia, and allodynia. One of the primary causes of these hypersensitivities is abnormal ectopic discharges and hyperexcitability that arise in the primary sensory neurons of the dorsal root ganglia (DRG) and neuromas at the sites of peripheral nerve injury (Chung and Chung, 2002; Campbell and Meyer, 2006; Devor, 2009; Wang et al., 2011). Nerve injury-induced changes in gene transcription and translation of receptors, enzymes, and ion channels in the DRG may contribute to this abnormal spontaneous activity (Zhao et al., 2013, 2017; Fan et al., 2014; Li et al., 2015; Liang et al., 2016a,b; Wu et al., 2016; Zhang et al., 2016; Sun L. et al., 2017). Thus, exploring how nerve injury drives the changes in gene transcription and translation in the DRG would open a new avenue in neuropathic pain treatment.

Recent studies revealed that DNA methylation, one type of epigenetic modification, participated in nerve injury-induced downregulation of pain-related genes in the DRG. Nerve injury-induced decreases in mu opioid receptor (MOR, encoded by *Oprm1* mRNA), kappa opioid receptor (KOR, encoding by *Oprk1* mRNA), and Kv1.2 (encoded by *Kcna2* mRNA) proteins may be attributed to an increase in the level of the DNA methyltransferase 3a protein (Dnmt3a) in the ipsilateral DRG neurons (Zhou et al., 2014; Sun L. et al., 2017; Zhao et al., 2017). Blocking this increase through DRG microinjection of adeno-associated virus type 5 (AAV5) expressing *Dnmt3a* shRNA prevented an elevation in DNA methylation within the promoter and 5′-untranslated region of *Oprm1* and *Kcna2* genes, and rescued the expression of *Oprm1* mRNA, *Oprk1* mRNA, *Kcna2* mRNA and their respective proteins in the ipsilateral DRG, restored morphine or loperamide (a peripheral acting MOR preferring agonist) analgesic effects, and attenuated nerve injury-induced pain hypersensitivity (Zhou et al., 2014; Sun L. et al., 2017; Zhao et al., 2017). Conversely, in the absence of nerve injury, mimicking this increase through DRG microinjection of AAV5 expressing full-length *Dnmt3a* reduced the expression of *Oprm1* mRNA*, Oprk1* mRNA*, Kcna2* mRNA, and their respective proteins, decreased Kv current and increased excitability in the DRG neurons, augmented MOR-controlled neurotransmitter release from the primary afferents, and led to spinal cord central sensitization, and neuropathic pain symptoms (Zhou et al., 2014; Sun L. et al., 2017; Zhao et al., 2017). These findings suggest that the increased Dnmt3a is a key player in neuropathic pain genesis through its participation in nerve injury-induced epigenetic silencing of the *Oprm1, Oprk1*, and *Kcna2* genes in the ipsilateral DRG. Therefore, it is essential to understand the molecular mechanism of how Dnmt3a is upregulated in the DRG following peripheral nerve injury.

MicroRNAs (miRs) are single-stranded, endogenous, small non-coding RNAs (18–25 nucleotides). They negatively regulate gene expression by recognizing the 3′-untranslated region (UTR) of target mRNA in a sequence-specific manner to post-transcriptionally inhibit the protein expression. Using *in silico* predictions, Dnmt3a was defined as a potential target of miR-143 (Ng et al., 2009). Ectopic expression of miR-143 in breast cancer cells or restoring expression of miR-143 in colorectal cancer cell lines repressed the Dnmt3a expression at both mRNA and protein levels (Ng et al., 2009, 2014). Moreover, Dnmt3a was demonstrated to be a direct target of miR-143 by luciferase reporter assay (Ng et al., 2009, 2014). Given that both miR-143 and Dnmt3a are expressed in the DRG neurons (Tam et al., 2011; Zhao et al., 2017), we proposed that miRA-143 might be involved in nerve injury-induced upregulation of Dnmt3a in the DRG under neuropathic pain conditions.

## Materials and methods

### Animal preparation

Male Sprague-Dawley rats weighing 200–220 g were purchased from Charles River Laboratories (Willington, MA). All animals were kept in a standard 12-h light/dark cycle, with water and food pellets available *ad libitum*. To minimize intra-individual and inter-individual variability of behavioral outcome measures, animals were acclimated for 2–3 days before behavioral testing was performed. All experiments conducted were approved by the Animal Care and Use Committee and Institutional Biosafety Committee at the Rutgers, the State University of New Jersey, and consistent with the ethical guidelines of the US National Institutes of Health and the International Association for the Study of Pain. All efforts were made in order to minimize animal suffering and to reduce the number of animals used. All of the experimenters were blind to surgical intervention and behavioral testing. No outliers were removed from the experiments.

### Neuropathic pain model

The fifth lumbar (L5) spinal nerve ligation (SNL)-induced neuropathic pain model in rats was carried out as described previously (Zhao et al., 2013; Li et al., 2015). Briefly, after rats were anesthetized with 2–3% isoflurane, the left L5 spinal nerves were isolated and ligated tightly with 4–0 silk suture under a surgical microscope. The ligated nerve was then transected at the distal end. Age-matched sham-treated control rats received identical surgical procedure without ligation and transection. The surgical field was finally irrigated with sterile saline and the skin incision was closed with wound clips.

### Morphine-induced analgesia

Morphine analgesia was measured in rats receiving subcutaneous (s.c) injection of 1.5 mg per kg morphine (WEST-WARD, Eatontown, NJ) on day 5 post-SNL or sham surgery. Paw withdrawal latencies to noxious heat, as described below, were measured before surgery (baseline latency) and 30 min after morphine injection (response latency). The cut-off latency is 20 s. The antinociceptive effects were expressed as the percentage of maximal possible analgesic effect (% MPAE): % MPAE = (response latency – baseline latency)/(cut-off latency - baseline latency)] × 100%. In addition, methylnaltrexone bromide (Medchemexpress USA, Monmouth Junction, NJ; a peripheral MOR antagonist, 5 mg/kg, dissolved in saline) or saline was intraperitoneally injected on day 5 post-SNL or sham surgery. Behavioral paw withdrawal responses to heat stimulation were carried out before microinjection, before SNL or sham surgery, and day 5 after SNL or sham surgery.

### Behavioral testing

Paw withdrawal thresholds in response to mechanical stimuli were measured with the up-down testing paradigm as described previously (Zhao et al., 2017). Briefly, the unrestrained rat was placed in a Plexiglas chamber on an elevated mesh screen. von Frey hairs in log increments of force (0.41, 0.69, 1.20, 2.04, 3.63, 5.50, 8.51, 15.14 g) were applied to the plantar surface of the rat's left and right hind paws. The 2.041-g stimulus was applied first. If a positive response occurred, the next smaller von Frey hair was used. If a negative response was observed, the next larger von Frey hair was used. The test ended when: (1) a negative response was obtained with the 15.14-g hair and (2) 3 stimuli were applied after the first positive response. Paw withdrawal threshold was determined by converting the pattern of positive and negative responses to the von Frey filament stimulation to a 50% threshold value with the formula provided by Dixon (Dixon, 1980).

Paw withdrawal latencies to noxious cold (0°C) were measured with a cold plate, the temperature of which was monitored continuously (Fan et al., 2014; Xu et al., 2014; Li et al., 2015). A differential thermocouple thermometer (Harvard Apparatus, South Natick, MA), attached to the plate, provided temperature precision of 0.1°C. Each rat was placed in a Plexiglas chamber on the cold plate, which was set at 0°C. The length of time between the placement of the hind paw on the plate and the animal jumping, with or without paw licking and flinching, was defined as the paw withdrawal latency. Each trial was repeated 3 times at 10-min intervals for each hind paw. A cutoff time of 60 s was used to avoid tissue damage of both hind paws.

Paw withdrawal latencies to noxious heat were measured with a Model 336 Analgesic Meter (IITC Inc./Life Science Instruments, Woodland Hills, CA) (Fan et al., 2014; Xu et al., 2014; Li et al., 2015). Rats were placed in a Plexiglas chamber on a glass plate. A radiant heat was applied by aiming a beam of light through a hole in the light box through the glass plate to the middle of the plantar surface of each hind paw. When the animal lifted its foot, the light beam was turned off. The length of time between the start of the light beam and the foot lift was defined as the paw withdrawal latency. Each trial was repeated 5 times at 5-min intervals for each side. A cut off time of 20 s was used to avoid tissue damage to the hind paw.

Locomotor function was examined according to the methods described previously (Park et al., 2009; Fan et al., 2014; Li et al., 2015). The following tests were performed before and after DRG microinjection; (1) Placing reflex: The rat was held with the hind limbs slightly lower than the forelimbs, and the dorsal surfaces of the hind paws were brought into contact with the edge of a table. The experimenter recorded whether the hind paws were placed on the table surface reflexively; (2) Grasping reflex: The rat was placed on a wire grid and the experimenter recorded whether the hind paws grasped the wire on contact; (3) Righting reflex: The rat was placed on its back on a flat surface and the experimenter noted whether it immediately assumed the normal upright position. Scores for placing, grasping, and righting reflexes were based on counts of each normal reflex exhibited in 5 trials (Park et al., 2009; Fan et al., 2014; Li et al., 2015). In addition, the rats' general behaviors, including spontaneous activity (e.g., walking and running), were observed.

### DRG neuronal culture and transfection

The 3 to 4 weeks old Sprague-Dawley rats were euthanized with isoflurane. All DRGs were collected in cold mixed Neurobasal medium (Gbico, Life technologies) containing 10% fetal bovine serum (FBS) (Gbico), 2% B27 supplement (Gbico), 1% L-glutamine (Gbico), and 1% antibiotics (100 U/ml penicillin and 100 μg/ml streptomycin; Gibco) and then treated with enzyme solution [dispase (3.5 mg/ml), collagenase type I (1.6 mg/ml) in Hanks' balanced salt solution without Ca^2+^ and Mg^2+^ (Gbico)]. After centrifugation, dissociated cells were suspended in mixed Neurobasal medium as described above and plated in a six-well plate coated with poly-d-lysine (0.5 mg/ml, Sigma, St. Louis, MO). The cells were incubated at 37°C in a humidified incubator with 5% CO_2_.

Chemically synthesized rat miR-143 mimics were used for the up-regulation of miR-143. Chemically modified antisense RNA molecules that optimized to specifically target endogenous miR-143 were used as miR-143 inhibitors. These RNAs and their respective negative controls (NC) were purchased from Ambion (Carlsbad, CA). AAV5 that expresses *Dnmt3a* shRNA (AAV5-3ashRNA) was used to specifically and selectively knock down *Dnmt3a* mRNA and Dnmt3a (Sun L. et al., 2017; Zhao et al., 2017). AAV5, which expresses enhanced green fluorescent protein (AAV5-GFP), was used as a control. One day after being plated, DRG cultured neurons were transfected with these oligonucleotides using Lipofectamine 2000 (Invitrogen) at the concentration of 100 nM according to the manufacturer's protocol or were transduced with 2 μl of AAV5 virus (titer ≥ 1 × 10^12^/ml). The neurons were collected 2 or 3 days later.

### Plasmid construction

The wild-type (WT) sequence of the rat *Dnmt3a* 3′-UTR containing the miR-143 binding site (186–192) was amplified from rat DRG cDNA using forward and reverse primers as shown in Table 1. To create the pmirGLO-Luc-*Dnmt3a* 3′-UTR WT vector, the resulting PCR fragment was cloned into the pmirGLO dual-luciferase miRNA target expression vector (Promega) using the XhoI and XbaI restriction sites (Promega). The mutant (MU) fragment contains three mutations in the “seed sequence” of the miR-143 binding site, which was synthesized using designed primers (Table 1) via overlap extension PCR and created a pmirGLO-Luc- Dnmt3a 3′-UTR MU vector. The sequences of all recombinant plasmids were confirmed by DNA sequencing.

**Table 1:** All primers used.

**Names**	**Sequences (5′ to 3′)**
**Real-time PCR**	
*miR-143* F	ACACTCCAGCTGGGTGAGATGAAGCACTGT
*miR-143* R	GGTGTCGTGGAGTCGGCAATTCAGTTGAG
*miR-143* RT	CTCAACTGGTGTCGTGGAGTCGGCAATTCAGTTGAGTGAGCTAC
*U6* F	CTCGCTTCGGCAGCACA
*U6* R and RT	AACGCTTCACGAATTTGCGT
*Dnmt3a* F	GTGGTTCGGAGATGGCAAAT
*Dnmt3a* R	TGGAGGACTTCGTAGATGGCT
*Oprm1* F	TTCCTGGTCATGTATGTGATTGTA
*Oprm1* R	GGGCAGTGTACTGGTCGCTAA
*Gapdh* F	TCGGTGTGAACGGATTTGGC
*Gapdh* R	CCTTCAGGTGAGCCCCAGC
**Cloning**	
*Dnmt3a* 3′-UTR F	GTACTCGAGAAGCAAACCACAGAGGAGGA
*Dnmt3a* 3′-UTR R	CAAGAGGTAACAGCGGCTTC
*Dnmt3a* 3′-UTR MU F	GGACATCA*GAC*CTTGAGTTTTC
*Dnmt3a* 3′-UTR MU R	TGAAAACTCAAG*GTC*TGATGTC

*F, Forward; R, Reverse; RT, Reverse-transcription; MU, mutant*.

### Luciferase reporter assay

PC-12 cells were prepared as described previously (Zhao et al., 2013, 2017). Briefly, the cells were cultured in Dulbecco's modified Eagle's medium/high glucose (Gibco) medium containing 5% FBS, 5% horse serum (Gibco), and 1% antibiotics. The cells with a confluency of ~60% were transfected with luciferase reporter plasmids and miR-143 mimics or negative control. Luciferase activity was assayed 48 h after transfection using the Dual-Luciferase Reporter Assay System (Promega). The ratio of firefly to *Renilla* luciferase was measured using a Centro XS3 LB 960 Microplate Luminometer (Berthold Technologies, Bad Wildbad, Germany). Transfection assays were repeated three times.

### DRG microinjection

Rat miR-143 mimics, inhibitors and their NCs were packed by TurboFect *in vivo* transfection reagent (Thermo Scientific Inc., Pittsburgh PA) to deliver RNA molecules to the DRG neuron as described before (Tan et al., 2005; Kawasaki et al., 2008; Xu et al., 2014). DRG microinjection was performed as described (Zhao et al., 2013, 2017; Cui et al., 2016; Zhang et al., 2016). In brief, a midline incision was made in the lower lumbar back region and the L4/5 or L5 DRG were exposed. The injected mixed solution (1 μl, 40 μM) was microinjected into 2 sites per DRG with a glass micropipette connected to a Hamilton syringe. The pipette was removed 10 min after injection. The surgical field was irrigated with sterile saline, and the skin incision was closed with wound clips. The injected rats showed no signs of paresis or other abnormalities. Consistent with previous reports (Zhao et al., 2013, 2017; Cui et al., 2016; Zhang et al., 2016), the injected DRG, stained with hematoxylin and eosin, retained its structural integrity and contained no visible leukocytes (Data not shown). The immune responses from microinjection were therefore minimal.

### RNA extraction and quantitative reverse transcription (qRT)-PCR

Total RNA was extracted from rat primary DRG cultured neurons or two pooled L5 DRGs from two individual rats using the miRNeasy Mini Kit (QIAGEN, Valencia, CA) according to manufacturer's instructions. RNA was reverse-transcribed using the ThermoScript reverse transcriptase (Invitrogen) with either the oligo (dT) primers (for *Dnmt3a, Oprml1*, and their internal control *Gapdh*) or specific reverse primers (for *miR-143* and its internal control *U6*). Primers for quantitative real-time PCR were listed in Table 1. Each sample was run in triplicate in a 20 μL reaction with 250 nM forward and reverse primers, 10 μL of SsoAdvanced Universal SYBR Green Supermix (Bio-Rad Laboratories, Hercules, CA) and 20 ng of cDNA. Reactions were performed in a BIO-RAD CFX96 real-time PCR system. The cycle parameters for miR-143 and U6 were as follows: 15 min incubation at 95°C, followed by 40 cycles of 95°C for 15 s, and 60°C for 1 min. The cycle parameters for the genes were as follows: 3 min incubation at 95°C, followed by 40 cycles of 95°C for 10 s, 60°C for 30 s, and 72°C for 30 s. Ratios of ipsilateral RNA to contralateral RNA were calculated by using the 2^−ΔΔCt^ method at a threshold of 0.02 as our pilot data indicated that the amplification reactions of the targeted genes and reference genes have similar PCR efficiency (Zhao et al., 2013). All targeted genes were normalized to the corresponding internal controls. Each experiment was repeated three times.

### Western blot analysis

The DRG was collected and homogenized with ice-cold lysis buffer [10 mM Tris, 5 mM ethylene glycol tetraacetic acid, 1 mM phenylmethylsulfonyl fluoride, 40 mM leupeptin, 5 mM magnesium chloride, and 1 mM dithiothreitol]. The crude homogenate was centrifuged at 4°C for 15 min at 1,000 g. The supernatant (membrane and cytosolic fractions) was collected for detecting MOR, ERK1/2, phospho-ERK1/2 (p-ERK1/2), GFAP, tubulin, and GAPDH. The pellet (nuclear fraction) was dissolved in lysis buffer containing 2% sodium dodecyl sulfate (SDS) and 0.1% Triton X-100 for detecting DMNT3a and H3. The proteins were separated on a 4–20% polyacrylamide gel (Bio-Rad) and transferred to a nitrocellulose membrane (Bio-Rad). The membranes were blocked with 3% non-fat milk in Tris-buffered saline containing 0.1% Tween-20 for 1 h and then incubated with primary antibodies overnight under gentle agitation. These antibodies included rabbit anti-DMNT3a (Cell Signaling Technology, Beverly, MA), rabbit anti-H3 (1:1,000, Cell Signaling Technology), mouse anti-MOR (1:500, Neuromics, Edina, MV), rabbit anti-p-ERK1/2 (Thr202/Tyr204, 1:1,000, Cell Signaling Technology), rabbit anti-ERK1/2 (1:1,000, Cell Signaling Technology), mouse anti-tubulin (1:1,000, Santa Cruz Biotechnology), rabbit anti-GFAP (1:1,000; Cell Signaling Technology), and rabbit anti-GAPDH (1:3,000, Santa Cruz Biotechnology). H3, tubulin, and GAPDH were used as internal loading controls. The proteins were detected by horseradish peroxidase-conjugated anti-mouse or anti-rabbit secondary antibody and visualized by Clarity Western ECL Substrate (Bio-Rad). The image signals were captured by a ChemiDoc imaging system and analyzed using Quantity One program (BioRad, Hercules, CA). The blot density from the control group was set as 100%. The relative density values from the other groups were determined by dividing the optical density values from these groups by the control value after each was normalized to the corresponding H3, tubulin, or GAPDH. Generally speaking, basal expression is expression without any treatments such as injection or surgery. Based on our previous studies (Zhao et al., 2013, 2017; Cui et al., 2016; Zhang et al., 2016), sham surgery or vehicle injection did not produce the expressional changes of the genes or proteins in the DRG. In addition, the expressional level in the contralateral DRG following SNL was not altered compared to that in the contralateral DRG of naive rats. Therefore, the expression level in the DRG from naive (0 day) rats, sham/vehicle treated group, or the contralateral side following SNL is considered as basal expression.

### Statistical analysis

All results are collected randomly and shown as mean ± SEM. After the normal distribution test, the data were analyzed using two-tailed, unpaired Student's *t-*test and a one-way or two-way ANOVA. When ANOVA showed a significant difference, pairwise comparisons between means were tested by the *post-hoc* Tukey method. *P* < 0.05 were considered statistically significant.

## Results

### Effect of rescuing miR-143 expression on SNL-induced upregulation of Dnmt3a mRNA and downregulation of Oprm1 mRNA in rat DRG

**Figure 1:**
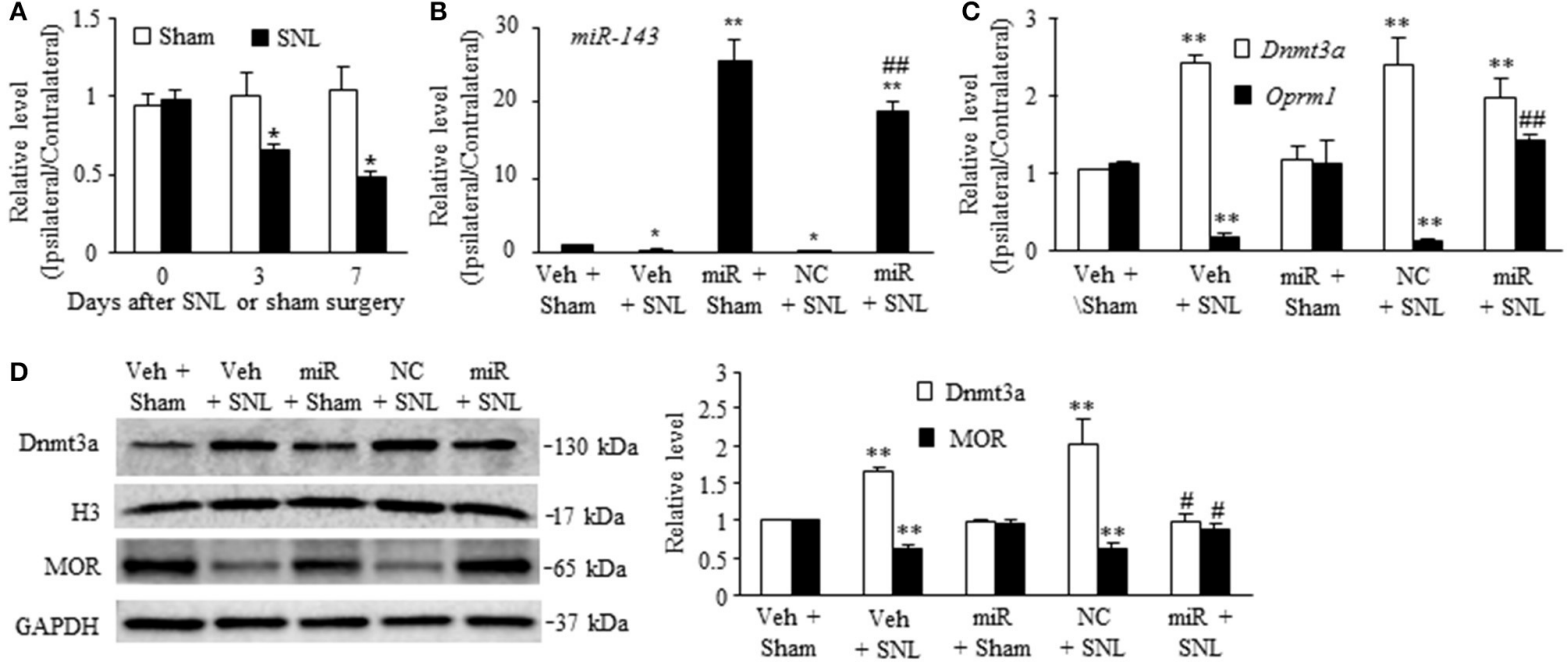
Rescuing miR-143 downregulation blocked the SNL-induced increase in Dnmt3a and restored the SNL-induced decreases in *Oprm1* mRNA and MOR in injured dorsal root ganglia (DRG). miR, microRNA; MOR, mu opioid receptor; NC, negative control; SNL, spinal nerve ligation, Veh, vehicle, **(A)** miR-143 decreased in the ipsilateral, but not contralateral, L5 DRG on days 3 and 7 after SNL, but not sham surgery. *n* = 6 rats/time points. Two-way ANOVA followed by Tukey *post-hoc* test. ^*^P < 0.05 vs. the corresponding naive group (0 day). **(B,C)** Microinjection of miR-143 mimics into the ipsilateral L5 DRG rescued the SNL-induced downregulation of miR-143 **(B)**, restored the SNL-induced decrease in *Oprm1* mRNA **(C)**, and did not affect the SNL-induced increase in *Dnmt3a* mRNA **(C)** in the ipsilateral L5 DRG on day 7 post-SNL. *n* = 6 rats/group. One-way ANOVA followed by Tukey *post-hoc* test. ^*^P < 0.05 or ^**^P < 0.01 vs. the corresponding vehicle plus sham group. ^*##*^*P* < 0.01 vs. the corresponding vehicle plus SNL group. **(D)** Microinjection of miR-143 mimics into the ipsilateral L5 DRG blocked the SNL-induced increase in Dnmt3a and restored SNL-induced decrease in MOR. Left, representative Western blots; Right, statistical summary of the densitometric analysis; *n* = 6 rats/group. One-way ANOVA followed by Tukey *post-hoc* test. ^**^P < 0.01 vs. the corresponding vehicle plus sham group. ^#^*P* < 0.05 vs. the corresponding vehicle plus SNL group.

To define the role of miR-143 in SNL-induced upregulation of *Dnmt3a* mRNA and its encoding Dnmt3a protein in the ipsilateral DRG, we first examined whether miR-143 expression was altered in the DRG after SNL. SNL time-dependently reduced the level of miR-143 in the L5 DRG on the ipsilateral, but not contralateral, side of rats (Figure 1A). The ratios of ipsilateral-side to contralateral-side of miR-143 were decreased by 34% on day 3 (*n* = 6 rats, *P* < 0.05) and 50% on day 7 (*n* = 6 rats, *P* < 0.05) post-SNL, compared to naive rats (0 day, *n* = 6 rats). As expected, sham surgery did not lead to any changes in basal levels of miR-143 in either ipsilateral or contralateral L5 DRG (Figure 1A). Neither SNL nor sham surgery affected relative expression of miR-143 in the ipsilateral and contralateral L4 DRG during the observation period (data not shown).

To further examine whether SNL-induced reduction of miR-143 was involved in the increases of *Dnmt3a* mRNA and Dnmt3a in the ipsilateral L5 DRG, we aimed to rescue miR-143 expression through microinjection of miR143 mimics into the ipsilateral L5 DRG. Negative control miRNA (NC, dissolved in saline) and vehicle (saline) were used as the controls. Given that nerve injury-induced epigenetic silencing of *Oprm1* mRNA was controlled by Dnmt3a in the DRG (Sun L. et al., 2017), the effects of microinjection of miR-143 mimics on SNL-induced decreases in DRG *Oprm1* mRNA and MOR were also observed. As expected, the level of miR-143 was significantly decreased in the ipsilateral L5 DRG on day 7 post-SNL in the rats microinjected with vehicle (*n* = 6 rats, *P* < 0.05. Figure 1B) or NC (*n* = 6 rats, *P* < 0.05; Figure 1B) and increased in the ipsilateral L5 DRG on day 7 post-sham surgery in the rats microinjected with miR-143 mimics (*n* = 6 rats, *P* < 0.01; Figure 1B). Microinjection of miR-143 mimics rescued the miR-143 expression demonstrated by a marked increase in the level of miR-143 in the ipsilateral L5 DRG on day 7 post-SNL (*n* = 6 rats, *P* < 0.01) as compared to the vehicle-treated sham rats (*n* = 6 rats) or SNL rats microinjected with vehicle or NC (Figure 1B). This rescue blocked the SNL-induced increase in the amount of Dnmt3a (*n* = 6 rats, *P* < 0.05), but not in the level of *Dnmt3a* mRNA (n = 6 rats, *P* > 0.05), in the ipsilateral L5 DRG on day 7 post-SNL and restored the expression of *Oprm1* mRNA (*n* = 6 rats, *P* < 0.01) and MOR (*n* = 6 rats, *P* < 0.05) evidenced by no reductions in the levels of *Oprm1* mRNA and MOR in the ipsilateral L5 DRG on day 7 post-SNL (Figures 1C,D). Unexpectedly, microinjection of siR-143 mimics did not affect relative expression of *Dnmt3a* RNA, *Oprm1* mRNA, and their respective proteins in the ipsilateral L5 DRG on day 7 post-sham surgery (*n* = 6 rats/group, *P* > 0.05. Figures 1C,D). Neither vehicle nor NC altered the SNL-induced increases in the levels of *Dnmt3a* mRNA and Dnmt3a and the SNL-induced decreases in the amounts of *Oprm1* mRNA and MOR in the ipsilateral L5 DRG on day 7 post-SNL (*n* = 6 rats/group, *P* > 0.05. Figures 1C,D).

### Effect of mimicking the SNL-induced decrease in miR-143 on relative expression of Dnmt3a and Oprm1 mRNAs and Dnmt3a and MOR proteins in the DRG

**Figure 2:**
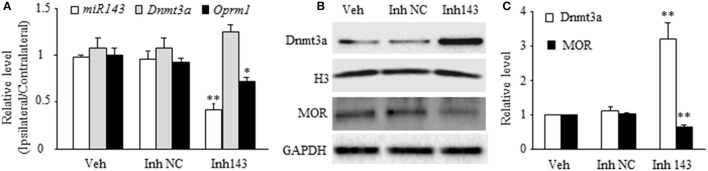
miR-143 knockdown caused by DRG microinjection of miR143 inhibitors increased the expression of Dnmt3a and reduced the expression of *Oprm1* mRNA and MOR in the injected DRG of naive rats. Inh143, miR-143 inhibitors; Inh NC, inhibitor negative control; Veh, vehicle. **(A)** Microinjection of miR-143 inhibitors, but not their negative control, into unilateral L4/5 DRGs reduced the expression of miR-143 and *Oprm1* mRNA and had no effect on relative expression of *Dnmt3a* mRNA. *n* = 3 6 rats/group. One-way ANOVA followed by Tukey *post-hoc* test. ^*^P < 0.05 or ^**^P < 0.01 vs. the corresponding vehicle group. **(B,C)** Microinjection of miR-143 inhibitors, but not their negative control, into unilateral L4/5 DRGs increased the expression of Dnmt3a and reduced the expression of MOR in the injected DRG. **(B)** representative Western blots. **(C)** statistical summary of the densitometric analysis. *n* = 6 rats/group. One-way ANOVA followed by Tukey *post-hoc* test. ^**^P < 0.01 vs. the corresponding vehicle group.

Next, we determined whether mimicking the SNL-induced decrease in DRG miR-143 through microinjection of miR-143 inhibitors into unilateral L4/5 DRGs affected relative expression of *Dnmt3a* mRNA, *Oprm1* mRNA, Dnmt3a, and MOR in the injected DRGs of naive rats. The miR-143 inhibitor negative control was used as a control. Microinjection of miR-143 inhibitors, but not the miR-143 inhibitor negative control, reduced the ratios of ipsilateral-side to contralateral-side of miR-143 by 57% (*n* = 6 rats, *P* < 0.01) and of *Oprm1* mRNA by 22% (*n* = 6 rats, *P* < 0.05) as compared to the corresponding vehicle-injected groups (*n* = 6 rats) on day 5 post-injection (Figure 2A). In addition, microinjection of miR-143 inhibitors decreased the amount of MOR by 37% (*n* = 6 rats, *P* < 0.01) compared to the vehicle-treated group in the injected DRGs on day 5 post-microinjection (Figures 2B,C). In contrast, the level of Dnmt3a was increased by 3.2-fold of the value of the vehicle-treated group (*n* = 6 rats, *P* < 0.01) in the injected DRG on day 5 post-injection (Figures 2B,C). Interestingly, microinjection of miR-143 inhibitors did not alter relative expression of *Dnmt3a* mRNA in the injected DRG on day 5 post-microinjection (*n* = 6 rats. Figure 2A).

### Expression of Dnmt3a and MOR regulated directly by miR-143 in DRG neurons

**Figure 3:**
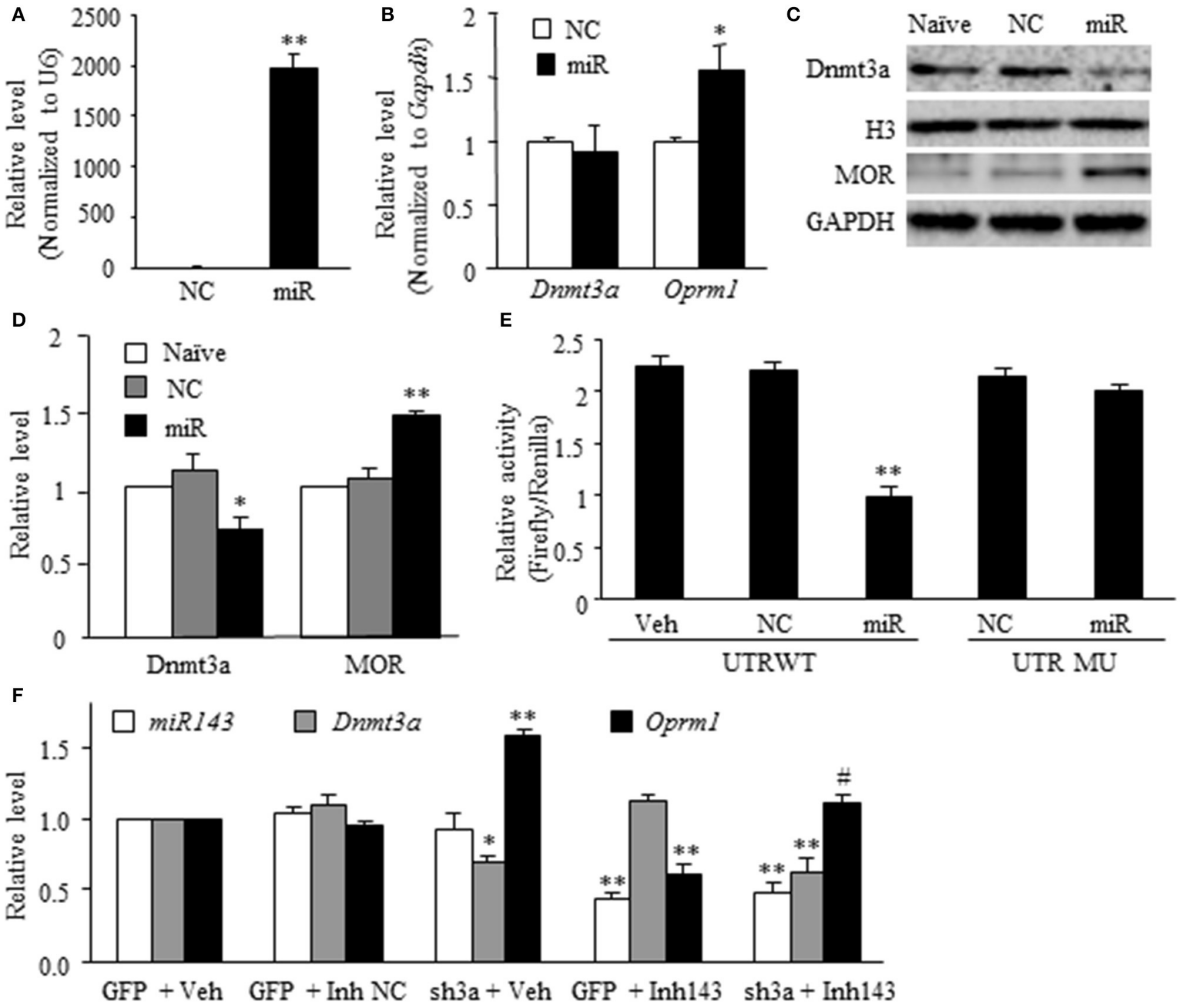
miR-143 directly regulates the expression of Dnmt3a and its downstream *Oprm1* mRNA and MOR in DRG neurons. miR, microRNA; NC, negative control; Veh, vehicle; **(A,B)** Transfection of miR-143 mimics (but not negative control) increased the expression of miR-143 **(A)** and *Oprm1* mRNA **(B)** and had no effect on the expression of *Dnmt3a* mRNA in the cultured DRG neurons. *n* = 3 repeats/group. Two-tailed, unpaired Student's *t-*test. ^*^P < 0.05 or ^**^P < 0.01 vs. the corresponding negative control group. **(C,D)** Transfection of miR-143 mimics (but not negative control) decreased the expression of Dnmt3a and increased the expression of *Oprm1* mRNA and MOR in the cultured DRG neurons. **(C)** representative Western blots. **(D)** Statistical summary of the densitometric analysis. *n* = 3 repeats/group. One-way ANOVA followed by Tukey *post-hoc* test. ^*^P < 0.05 or ^**^P < 0.01 vs. the corresponding naive group. **(E)** Luciferase reporter assay revealed that transfection of miR-143 mimics, but not negative control, decreased translational activity in the 3′untranslated region (UTR) of *Dnmt3a* mRNA containing the miR-143 binding sites (UTR WT) and failed to affect basal luciferase activity in the mutant 3′-UTR of *Dnmt3a* mRNA in which the miR-143 binding sites was mutated (UTR MU). *n* = 3 repeats/group. One-way ANOVA followed by Tukey *post-hoc* test. ^**^P < 0.01 vs. the corresponding vehicle group. **(F)** Transfection of miR-143 inhibitors (Inh143), but not inhibitor negative control (Inh NC), reduced the expression of miR-143 and *Oprm1* mRNA and had no effect on the expression of *Dnmt3a* mRNA in the cultured DRG neurons. The miR-143 inhibitors-induced reduction in *Oprm1* mRNA was completely reversed by co-administration of AAV5 expressing *Dnmt3a* shRNA (sh3a). *n* = 3 repeats/group. One-way ANOVA followed by Tukey *post-hoc* test. ^*^P < 0.05 or ^**^P < 0.01 vs. the corresponding GFP (enhanced green fluorescent protein) plus vehicle group. ^#^*P* < 0.05 vs. the corresponding GFP plus Inh143 group.

The *in vivo* work described above could not tell whether miR-143 directly regulated the expression of Dnmt3a and MOR in the DRG neurons. To this end, the best model might be the dissociated DRG neuronal culture from adult rats. We first examined whether miR-143 truly affected the expression of Dnmt3a and MOR in the cultured DRG neurons. The transfection of miR-143 mimics increased the levels of miR-143 and *Oprm1* mRNA, respectively, by 1,965-flod (*n* = 3 repeats, *P* < 0.01. Figure 3A) and 1.56-fold (*n* = 3 repeats, *P* < 0.05. Figure 3B) compared to the negative control and increased the amount of MOR by 1.48-fold (*n* = 3 repeats, *P* < 0.01. Figures 3C,D) compared to naive group. This transfection did not affect the expression of *Dnmt3a* mRNA (*n* = 3 repeats, *P* > 0.05. Figure 3B), but reduced the level of Dnmt3a protein by 23% (*n* = 3 repeats, *P* < 0.05, Figures 3C,D) compared to naive group. This finding, combined with our *in vivo* observations above, suggests the post-transcriptional inhibition of *Dnmt3a* mRNA occurred in the presence of miR-143. To further test this conclusion, we then carried out luciferase reporter assay and found that the transfection of miR-143 mimics significantly reduced the translational activity in the 3′-UTR of *Dnmt3a* mRNA containing the miR-143 binding site (*n* = 3 repeats, *P* < 0.01), but not in the mutant 3′-UTR of *Dnmt3a* mRNA, in which the miR-143 binding site was mutated (Figure 3E). Finally, we determined whether Dnmt3a was required for miR-143 regulation of the expression of *Oprm1* mRNA in the DRG neurons. shRNA strategy was used to knockdown DRG Dnmt3a expression through transduction of AAV5-3ashRNA into the cultured DRG neurons (Sun L. et al., 2017; Zhao et al., 2017). AAV5-GFP was used as a control. Consistent with the previous report(Sun L. et al., 2017), AAV5-3ashRNA, but not AAV5-GFP, knocked down *Dnmt3a* mRNA (*n* = 3 repeats, *P* < 0.05) and significantly increased the expression of *Oprm1* mRNA (*n* = 3 repeats, *P* < 0.01) in the cultured DRG neurons (Figure 3F). As expected, the miR-143 inhibitors, but bot the inhibitor negative control, produced the decreases not only in the expression of miR-143 (*n* = 3 repeats, *P* < 0.01) but also in the expression of *Oprm1* mRNA (*n* = 3 repeats, *P* < 0.01), although the inhibitor did not affect relative expression of *Dnmt3a* mRNA in the AAV5-GFP-tranduced DRG neurons (Figure 3F). The inhibitor-induced decrease of *Oprm1* mRNA could be completely reversed via co-administration of AAV5-3ashRNA (*n* = 3 repeats, *P* < 0.05. Figure 3F). These findings suggest that miR-143 directly and negatively regulates post-transcriptional expression of *Dnmt3a* mRNA, resulting in Dnmt3 protein downregulation and subsequently disinhibition of the downstream *Oprm1* mRNA expression in the DRG neurons.

### Effect of rescuing DRG miR-143 expression on SNL-induced pain hypersensitivities

**Figure 4:**
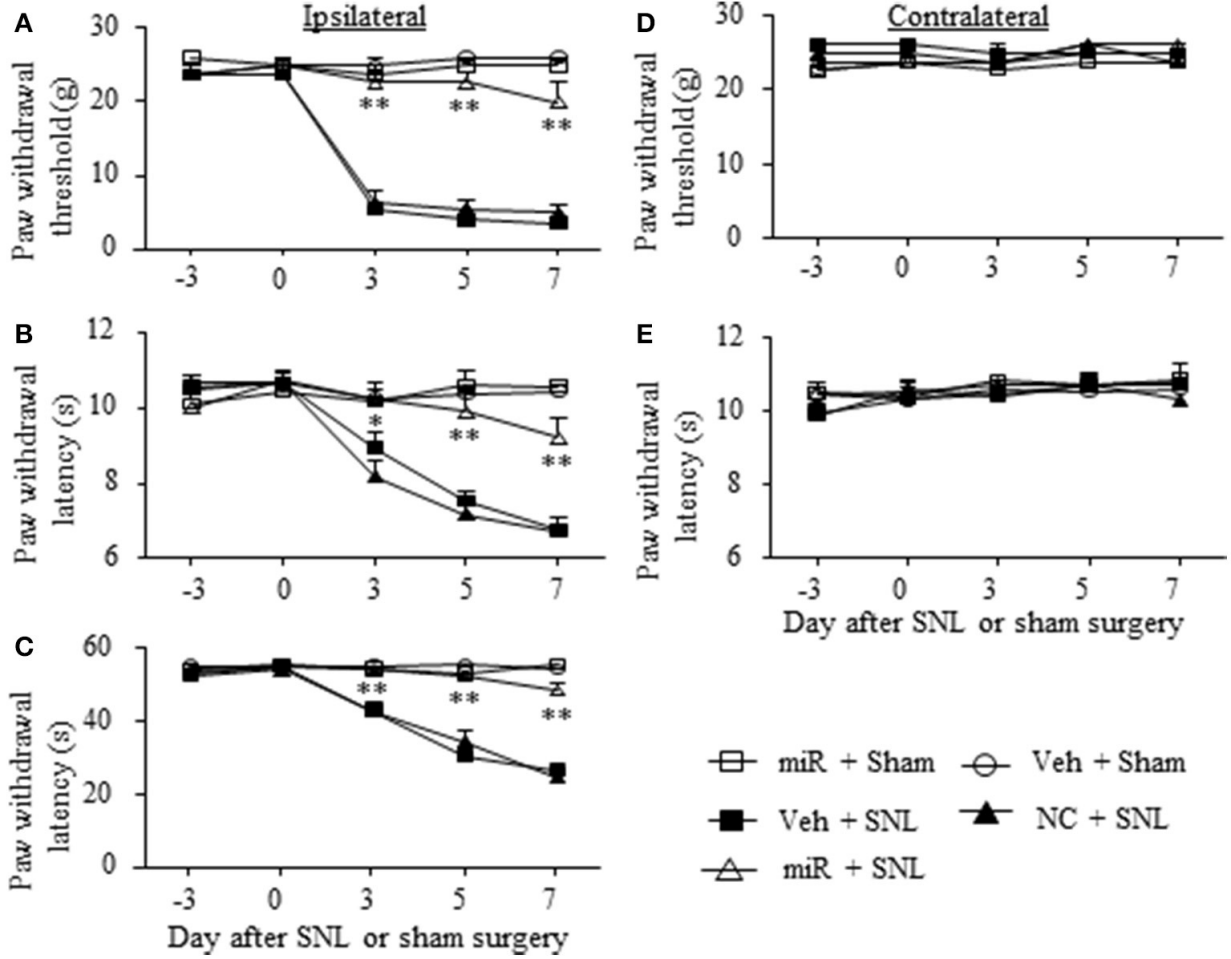
Rescuing DRG miR-143 expression impaired neuropathic pain. miR, microRNA; NC, negative control; SNL, spinal nerve ligation; Veh, vehicle; Microinjection of miR-143 mimics, but not negative control, into the ipsilateral L5 DRG significantly attenuated SNL-induced decreases in paw withdrawal threshold in response to mechanical stimulation **(A)**, in paw withdrawal latency in response to thermal stimulation **(B)** and in paw withdrawal jump latency in response to cold stimulation **(C)** on the ipsilateral side on days 3, 5, and 7 after SNL. Neither miR-143 mimics nor negative control altered paw responses to mechanical **(D)** and thermal **(E)** stimuli on the contralateral side. No changes in paw responses were seen on either ipsilateral or contralateral side in sham rats microinjected with vehicle or miR-143 mimics. *n* = 5 rats/group. Two-way ANOVA followed by Tukey *post-hoc* test. ^*^P < 0.05 or ^**^P < 0.01 vs. the vehicle plus SNL group at the corresponding time point.

Given that miR-143 is likely a negative regulator in DRG Dnmt3a expression under neuropathic pain conditions and that Dnmt3a acts as an endogenous contributor to neuropathic pain genesis (Sun L. et al., 2017; Zhao et al., 2017), we proposed that SNL-induced reduction of DRG miR-143 might participate in the development of SNL-induced pain hypersensitivities. To address our proposal, we examined the effect of rescuing miR-143 expression in the ipsilateral DRG on SNL-induced mechanical allodynia, thermal hyperalgesia, and cold allodynia. Consistent with previous studies (Zhao et al., 2013, 2017; Li et al., 2015), SNL produced long-term mechanical allodynia, thermal hyperalgesia, and cold allodynia on the ipsilateral side in the vehicle-injected rats (Figures 4A–C). The paw withdrawal thresholds in response to mechanical stimulation applied to the ipsilateral hind paw were significantly decreased on day 3 (*P* < 0.01), 5 (*P* < 0.01), and 7 (*P* < 0.01) post-SNL as compared with preinjury baseline values (*n* = 5 rats. Figure 4A). The paw withdrawal latency and jump latency of the ipsilateral hind paw in response to heat and cold, respectively, were markedly reduced on day 3 (*P* < 0.05), 5 (*P* < 0.01) and 7 (*P* < 0.01) post-SNL as compared to the corresponding baseline (*n* = 5 rats. Figures 4B,C). Microinjection of miR-143 mimics did not change paw responses to mechanical, heat, or cold stimuli on the ipsilateral side of sham rats (*n* = 5 rats. Figures 4A–C), but microinjection of miR-143 mimics abolished SNL-induced mechanical allodynia, thermal hyperalgesia, and cold allodynia (Figures 4A–C). Compared with the baseline values, there were no significant changes in paw withdrawal thresholds and latencies and paw jumping latencies on the ipsilateral side of the miR-143 mimics-injected SNL rats (*n* = 5 rats. Figures 4A–C). As expected, microinjection of miR-143 negative control did not affect SNL-induced mechanical allodynia, thermal hyperalgesia, and cold allodynia on the ipsilateral side during the observation period (*n* = 5 rats. Figures 4A–C). There were no marked differences in paw responses between the negative control-microinjected and vehicle-microinjected groups (Figures 4A–C). Microinjection of neither miR-143 mimics, negative control, nor vehicle altered basal paw responses on the contralateral side (Figures 4D,E) and locomotor function (Table 2).

**Table 2:** Mean (*SD*) changes in locomotor function.

**Group**	**Functional test**		
	**Placing**	**Grasping**	**Righting**
Vehicle + Sham	5 (0)	5 (0)	5 (0)
Vehicle + SNL	5 (0)	5 (0)	5 (0)
NC + SNL	5 (0)	5 (0)	5 (0)
miR-143 + Sham	5 (0)	5 (0)	5 (0)
miR-143 + SNL	5 (0)	5 (0)	5 (0)
Vehicle	5 (0)	5 (0)	5 (0)
miR-143 inhibitors	5 (0)	5 (0)	5 (0)
Inhibitor NC	5 (0)	5 (0)	5 (0)

*n = 5 rats/group. 5 trials. miR, microRNA; NC, negative control; SNL, spinal nerve ligation*.

**Figure 5:**
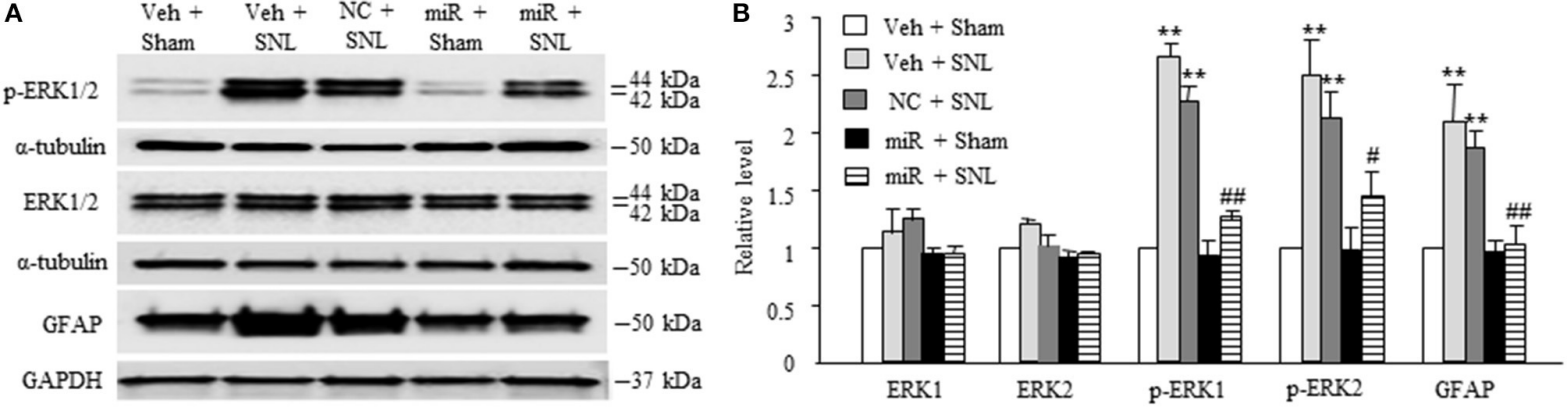
Rescuing DRG miR-143 expression alleviated central sensitization in spinal dorsal horn. miR, microRNA; NC, negative control; SNL, spinal nerve ligation; Veh, vehicle; Microinjection of miR-143 mimics, but not negative control, into the ipsilateral L5 DRG significantly attenuated SNL-induced increases in the levels of phosphorylated extracellular signal-regulated kinases ½ (p-ERK1/2) and glial fibrillary acidic protein (GFAP) in the ipsilateral L5 dorsal horn on day 7 post-SNL. **(A)** representative Western blots. **(B)** statistical summary of the densitometric analysis. *n* = 6 rats/group. One-way ANOVA followed by Tukey *post-hoc* test. ^**^P < 0.01 vs. the corresponding vehicle plus sham group. ^#^*P* < 0.05 or ^*##*^*P* < 0.01 vs. the corresponding vehicle plus SNL group.

We also examined whether microinjection of miR-143 mimics altered SNL-induced dorsal horn central sensitization indicated by the increases in phosphorylated-extracellular signal–regulated kinase 1/2 (p-ERK1/2) and glial fibrillary acidic protein (GFAP) in dorsal horn (Latremoliere and Woolf, 2009; Zhang et al., 2016). The amounts of phosphorylated-ERK1/2 (not total ERK1/2) and GFAP were significantly increased in the ipsilateral L5 dorsal horn in rats subjected to SNL (*n* = 6 rats, *P* < 0.01) but not in those that received sham surgery and vehicle (*n* = 6 rats. Figures 5A,B). These increases were significantly blocked in the miR-143 mimics-microinjected SNL rats (Figures 5A,B).

### Effect of mimicking the SNL-induced decrease in DRG miR-143 on basal nociceptive thresholds in naive rats

**Figure 6:**
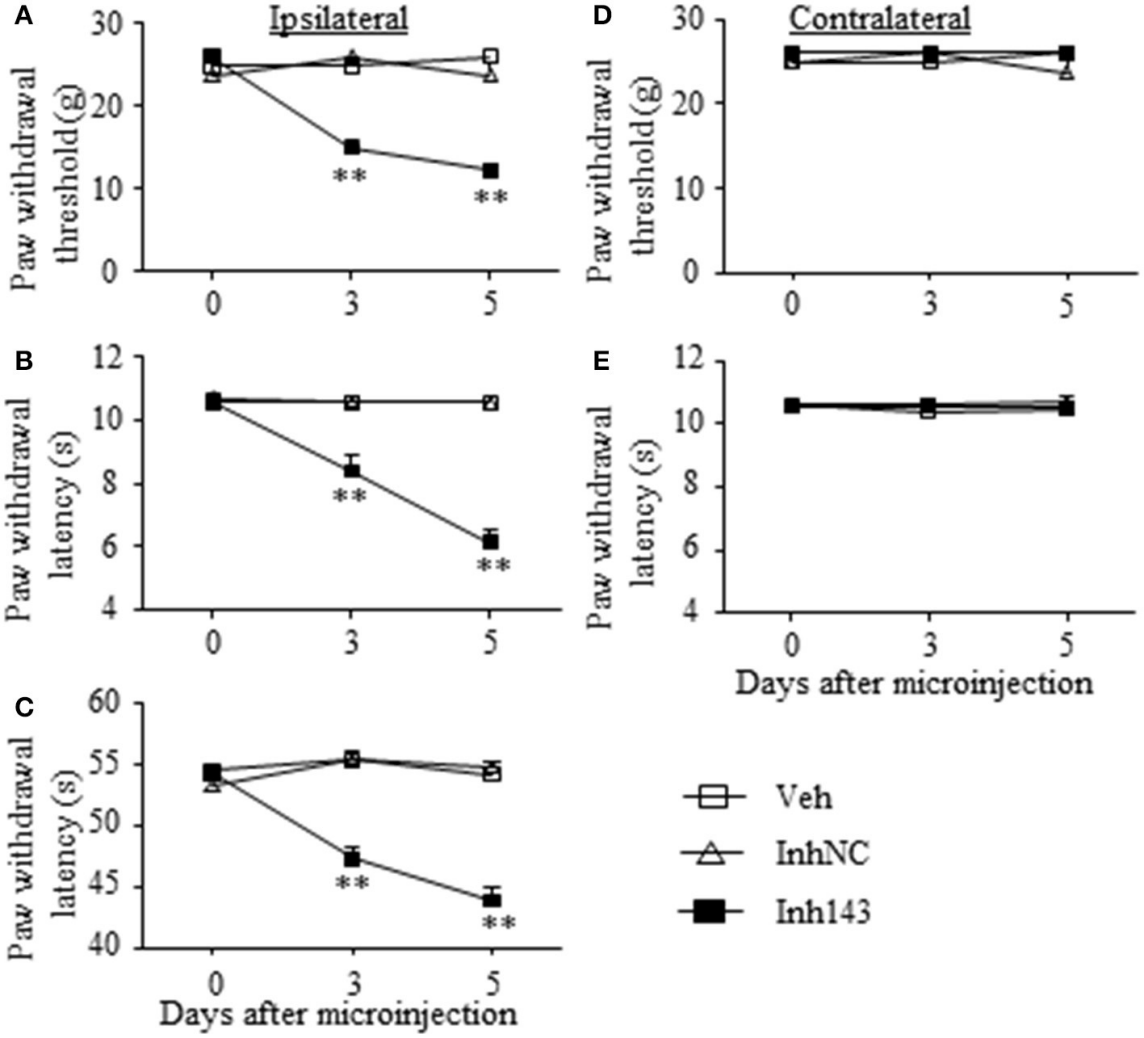
miR-143 knockdown caused by DRG microinjection of miR-143 inhibitors produced neuropathic pain-like symptoms in naive rats. Paw withdrawal responses to mechanical **(A,D)**, thermal **(B,E)** and cold **(C)** stimuli on the ipsilateral **(A–C)** and contralateral **(D,E)** sides from the treated groups as shown. Inh143, miR-143 inhibitors; Inh NC, inhibitor negative control; Veh, vehicle; *n* = 5 rats/group. Two-way ANOVA followed by Tukey *post-hoc* test. ^**^P < 0.01 vs. the vehicle group at the corresponding time point.

We further determined whether the SNL-induced decrease in DRG miR-143 was sufficient for SNL-induced pain hypersensitivities. Mimicking SNL-induced decrease in DRG miR-143 through microinjection of miR-143 inhibitors into the unilateral L4/5 DRGs led to mechanical allodynia as demonstrated by ipsilateral decrease in paw withdrawal threshold in responses to mechanical stimulation (*n* = 5 rats, *P* < 0.01. Figure 6A). Microinjection of miR-143 inhibitors also produced thermal hyperalgesia and cold allodynia as evidenced by ipsilateral decreases in paw withdrawal latencies in response to heat stimulation (*n* = 5 rats, *P* < 0.01. Figure 6B) and paw jumping latencies to cold stimulation, respectively (*n* = 5 rats, *P* < 0.01. Figure 6C). These pain hypersensitivities developed on day 3 post-microinjection and lasted for at least 5 days after microinjection (Figures 6A–C). No significant changes in basal paw withdrawal responses to mechanical and thermal stimuli on the contralateral side (Figures 6D,E) and locomotor function (Table 2) were seen in the rats microinjected with miR-143 inhibitors. As expected, microinjection of neither vehicle nor miR-143 inhibitor negative control markedly altered baselines in the response to mechanical, thermal, and cold stimuli on both ipsilateral and contralateral sides (Figures 6A–E) and locomotor function (Table 2).

### Effect of rescuing DRG miR-143 expression on morphine analgesia after SNL

**Figure 7:**
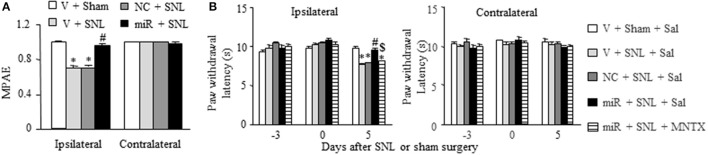
Rescuing DRG miR-143 expression improved morphine analgesia under neuropathic pain conditions. miR, microRNA; NC, negative control; SNL, spinal nerve ligation; Veh, vehicle; **(A)** Microinjection of miR-143 mimics, but not negative control, into the ipsilateral L5 DRG reversed the decrease in morphine analgesia on the ipsilateral side 5 days after SNL. MPAE, maximal possible analgesic effect; *n* = 5 rats/group. One-way ANOVA followed by Tukey *post-hoc* test. ^*^P < 0.05 vs. the corresponding vehicle plus sham group. ^#^*P* < 0.05 vs. the corresponding vehicle plus SNL group. **(B)** Intraperitoneal injection with methylnatrexone (MNTX) blocked the miR-143 mimics-induced antinociception on day 5 post-SNL in the ipsilateral side of the miR-143 mimics plus SNL group. MNTX was administered on day 5 post-SNL or sham surgery. Behavioral tests were carried out 30 min after drug administration. *n* = 5 rats/group. Two-way ANOVA followed by Tukey *post-hoc* test. ^*^P < 0.05 vs. the vehicle plus sham plus saline (Sal) group at the corresponding time point. ^#^*P* < 0.05 vs. the vehicle plus SNL plus saline group at the corresponding time point. ^$^*P* < 0.05 vs. the miR-143 mimics plus SNL plus saline group at the corresponding time point.

Finally, we examined whether rescuing DRG miR-143 expression improved morphine analgesia under SNL-induced neuropathic pain conditions. Consistent with previous studies (Rashid et al., 2004; Zhou et al., 2014; Zhang et al., 2016), morphine analgesia on day 3 post-SNL significantly reduced compared to that on day 3 post-sham surgery on the ipsilateral side of the vehicle-microinjected group (*n* = 5 rats, *P* < 0.05. Figure 7A). This reduction was reversed markedly on the ipsilateral side of miR-143 mimics-microinjected rats (*n* = 5 rats, *P* < 0.05), but not in negative control-treated rats (*n* = 5 rats. Figure 7A). As expected, morphine produced robust analgesia on the contralateral side of all treated groups (Figure 7A). Additionally, in line with our observations in Figure 4C, microinjection of miR-143 mimics into the ipsilateral L5 DRG significantly reversed the SNL-induced decrease in paw withdrawal latency in response to thermal stimulation on the ipsilateral side following intraperitoneal saline injection (*n* = 5 rats, *P* < 0.05. Figure 7B). However, this effect was absent after intraperitoneal methylnaltrexone administration (*n* = 5 rats, *P* < 0.05. Figure 7B). Methylnaltrexone at the dose used did not affect basal behavioral responses on the contralateral side (Figure 7B). Taken together, these findings further demonstrated the implication of miR-143 in nerve injury-induced MOR downregulation in the ipsilateral DRG.

## Discussion

Neuropathic pain has been intensively studied, but how peripheral nerve injury causes pain hypersensitivities is still incompletely understood. Recent evidence revealed that epigenetic modification, such as DNA methylation, plays a key role in neuropathic pain genesis (Lutz et al., 2014; Liang et al., 2015). Peripheral never injury-induced increase in DRG Dnmt3a expression contributes to neuropathic pain by elevating DNA methylation in the promoter and 5′-UTR of pain-associated genes (such as *Oprm1, Oprk1*, and *Kcna2*) and subsequently, repressing their expression in the primary sensory neurons (Zhou et al., 2014; Sun L. et al., 2017; Zhao et al., 2017). In the present study, we demonstrated that this increase was attributed at least in part to peripheral nerve injury-induced downregulation of miR-143 in the DRG. miR-143 likely participates in the mechanisms that underlie neuropathic pain.

Peripheral nerve injury upregulates DRG Dnmt3a expression through multiple mechanisms. We previously reported that the octamer transcription factor (OCT1) promoted DRG *Dnmt3a* gene activity after SNL (Zhao et al., 2017). OCT1 specifically binds to a consensus binding motif in the promoter of the *Dnmt3a* gene (Zhao et al., 2017). SNL-induced increase in DRG OCT1 expression enhanced the binding activity of OCT1 to the *Dnmt3a* gene, resulting in the promotion of transcription and translation of *Dnmt3a* mRNA in the injured DRG (Zhao et al., 2017). However, whether the increased OCT1 in the DRG participated in neuropathic pain is still unknown and remains to be further investigated. The present study showed that SNL-induced downregulation of miR-143 was also required for SNL-induced upregulation of Dnmt3a in the injured DRG. Our *in vitro* and *in vivo* experiments revealed that either miR-143 mimics or its inhibitors did not affect the expression of *Dnmt3a* mRNA, but altered the expression of Dnmt3a protein in the DRG neurons. These findings suggest that miR-143 negatively and post-transcriptionally regulates the expression of Dnmt3a through binding to the 3′-UTR of *Dnmt3a* mRNA and suppressing *Dnmt3a* mRNA translation in the DRG. This conclusion is supported by the fact that most animal miRNAs inhibit protein synthesis through an unknown mechanism that preserves the stability of the mRNA target (Ambros, 2004). Interestingly, miR-143 in the breast and colorectal cancer tissues affected the Dnmt3a expression at both mRNA and protein levels (Ng et al., 2009, 2014). The reason why miR-143 had distinct effects on *Dnmt3a* mRNA expression between the present study and the previous reports (Ng et al., 2009, 2014) is unknown but may be related to the tissue difference. We also noticed that DRG microinjection of miR-143 mimics did not alter relative expression of Dnmt3a in sham rats. However, transfection of miR-143 mimics into the cultured DRG neurons significantly reduced the expression of Dnmt3a. No effect of miR-143 mimics on *in vivo* Dnmt3a expression may be due to the low level of Dnmt3a expression in the DRG under normal conditions. miR-143 mimics at the dose injected could not further reduce basal level of Dnmt3a expression in the sham rats, despite the fact that miR-143 mimics at this dose markedly blocked the SNL-induced increase in Dnmt3a expression in the injured DRG. It is worth noting that, in addition to OCT1 and miR143, whether additional transcription factors and/or microRNAs are involved in nerve injury-induced upregulation of DRG Dnmt3a remains to be determined.

The role of microRNAs in neuropathic pain has been documented, but the mechanisms of how they contribute to this disorder are still elusive (Lutz et al., 2014; Sakai and Suzuki, 2014). Previous studies reported that specific microRNAs could alleviate neuropathic pain development through targeting specific proteins in the DRG or spinal dorsal horn (Chen et al., 2014; Liu et al., 2015; Tan et al., 2015; Sun W. et al., 2017; Yan et al., 2017). The present study demonstrated that SNL-induced downregulation of DRG miR-143 contributed to SNL-induced pain hypersensitivities through post-transcriptional disinhibition of Dnmt3a expression, resulting in its upregulation and subsequent downregulation of its downstream target *Oprm1* mRNA, in the injured DRG. Dnmt3a, an epigenetic repressor, participates in epigenetic silencing of several genes (such as *Oprk1* and *Kcna2* besides *Oprm1*) in the injured DRG following peripheral nerve injury (Sun L. et al., 2017; Zhao et al., 2017). Moreover, besides Dnmt3a, miR-143 may potentially and post-transcriptionally inhibit the expression of other genes. Therefore, miR-143 is implicated in neuropathic pain likely through regulating the expression of multiple targeting genes in the DRG.

In summary, our study revealed that miR-143 downregulation participated in a Dnmt3a-triggered epigenetic mechanism of *Oprm1* mRNA decrease in the injured DRG under neuropathic pain conditions. Given that Dnmt3a and *Oprm1* are key players in neuropathic pain development (Sun L. et al., 2017; Zhao et al., 2017) and that miR-143 mimics impaired this disorder without changing locomotor function and acute pain, miR-143 may be a target for neuropathic pain management. Nevertheless, miR-143 overexpression by its mimics may cause side effects as miR-143 has multiple downstream targets as discussed above.

## Author contributions

Y-XT conceived the project and supervised all experiments. BX, JC, JZ, SJ, SW, and Y-XT designed the project. BX, JC, JZ, SJ, SW, LL, KM, and XM performed molecular, biochemical, and behavioral experiments. BX, JC, JZ, SJ, SW, AB, and Y-XT analyzed the data. Y-XT wrote the manuscript. All of the authors read and discussed the manuscript.

### Conflict of interest statement

The authors declare that the research was conducted in the absence of any commercial or financial relationships that could be construed as a potential conflict of interest.
