# Corrigendum: Role of MicroRNA-143 in Nerve Injury-Induced Upregulation of Dnmt3a Expression in Primary Sensory Neurons

**DOI:** 10.3389/fnmol.2020.599615

**Published:** 2020-10-22

**Authors:** Bo Xu, Jing Cao, Jun Zhang, Shushan Jia, Shaogen Wu, Kai Mo, Guihua Wei, Lingli Liang, Xuerong Miao, Alex Bekker, Yuan-Xiang Tao

**Affiliations:** ^1^Department of Anesthesiology, Rutgers New Jersey Medical School, The State University of New Jersey, Newark, NJ, United States; ^2^Department of Anesthesiology, General Hospital of Guangzhou Military Command, Guangzhou, China; ^3^Neuroscience Research Institute, College of Basic Medicine, Zhengzhou University, Zhengzhou, China; ^4^Department of Anesthesiology, Union Medical Center, Tianjin, China; ^5^Department of Anesthesiology, Yantai Affiliated Hospital of Binzhou Medical University, Yantai, China; ^6^Departments of Cell Biology & Molecular Medicine and Physiology, Pharmacology & Neuroscience, Rutgers New Jersey Medical School, The State University of New Jersey, Newark, NJ, United States

**Keywords:** miR-143, Dnmt3a, *Oprm1*, dorsal root ganglion, neuropathic pain

In the published article, there was an error in affiliation for Yuan-Xiang Tao. Instead of having affiliations 1, 3, 6, they should have affiliations 1, 6.

The authors apologize for this error and state that this does not change the scientific conclusions of the article in any way. The original article has been updated.

